# Rare Presentation of Self-Limiting Kikuchi–Fujimoto Disease in Relapsing Nature

**DOI:** 10.1155/2020/9785104

**Published:** 2020-09-21

**Authors:** Karo Servatyari, Hero Yazdanpanah, Chamara Dalugama

**Affiliations:** ^1^Student Research Committee, Kurdistan University of Medical Sciences, Sanandaj, Iran; ^2^Lecturer in Medicine, Department of Medicine, University of Peradeniya, Peradeniya, Sri Lanka

## Abstract

**Background:**

Kikuchi–Fujimoto disease (KFD) is a rare, benign, and self-limiting disease that is commonly associated with cervical lymphadenopathy and fever. The disease has a wide spectrum of clinical manifestations, and definitive diagnosis is based on the histological appearance in the excision biopsy of the lymph nodes. Recurrence of KFD is reported rarely. *Case Presentation*. A 56-year-old Iranian woman with a background history of thrombocytopenia presented with fever, malaise, loss of appetite, and weight loss with cervical lymphadenopathy. The excision biopsy of the cervical lymph nodes confirmed the diagnosis of KFD, and she made a full recovery with improvement of symptoms, regression of cervical lymph nodes, and normalization inflammatory markers. One year after remission, she presented with similar clinico-biochemical profile, and repeat biopsy confirmed KFD.

**Conclusion:**

Although the rate of recurrence of the disease is very low, the treating physician should consider the possibility and confirm it histologically.

## 1. Introduction

Kikuchi necrotizing lymphadenitis is a rare, benign, and self-limiting disease that is commonly associated with cervical lymphadenopathy and fever [1, 2]. Many reports suggest a possible link to a variety of microorganisms, from bacteria to viruses [3, 4]. KFD is generally reported more common in women than men [5, 6]. The average age of people involved is less than 40 years and is more common in young people [6]. Skin rash, arthritis, fatigue, and hepatosplenomegaly are reported [7].The definitive diagnosis of this disease is made by excisional biopsy of the cervical lymph nodes and is based on histopathology [8]. However, it is possible to approach the diagnosis in special cases with a skilled radiologist with specific computed tomography appearance of the involved lymph node groups [9]. Most cases of KFD are self-limiting, but some cases are treated with steroids, NSAIDs, and immunoglobulins with variable success [10–13].

The recurrence rate of the disease is rare and is about 3–4% [14]. In a case of recurrent symptoms with lymphadenopathy, possibility of recurrence of KFD needs to be considered and actively excluded histologically as other sinister possibilities include infections or malignancy.

## 2. Case Presentation

A 56-year-old Iranian woman presented with a one-month history of low-grade fever with generalized malaise, loss of weight, and loss of appetite, and she had noticed swelling of neck glands for the similar duration. She denied any joint pains, increased hair loss, or oral ulcers. She did not have chronic cough, sore throat, or ear discharge. The patient denied any alteration of bowel habits or melena. She denied any past history and contact history of tuberculosis or any high-risk sex behaviours. Her past medical history was significant for hypertension and dyslipidemia. One month prior to this presentation, she was seen by a rheumatologist with inflammatory bilateral knee joint arthritis. Her investigations were significant for thrombocytopenia of 109 × 10^9^/L (150–400 × 10^9^/L). There was no involvement of other cell lines. In the workup for the ANA, rheumatoid factor and anti-CCP were negative.

On examination, she was febrile. She was neither pale nor icteric. She had firm, tender discrete lymphadenopathy, the largest measuring 2 cm, in the right upper cervical region. Respiratory and cardiovascular system examinations were normal. Abdominal examination was normal without evidence of organomegaly.

Neck ultrasonography revealed multiple cervical lymph nodes in the cervical region, the largest of which was 24 mm in size in the right submandibular, as well as numerous lymphadenopathies were seen in the right axillary region. Her blood investigations are summarized in Table 1.

The patient was treated with a course of oral antibiotics considering the possibility of bacterial lymphadenopathy (co-amoxiclav and metronidazole). No improvement in symptomatology was observed over 7-day course of antibiotics, and in addition, she was noted to have a generalized urticarial skin rash.

She underwent excision biopsy of the cervical lymph node. Histopathology revealed areas of necrosis, infiltrated with nuclear dust, paucity of granulocytes, and surrounding tissue showing mononuclear cells and reported as “benign acute necrotizing lymphadenitis” which was compatible with Kikuchi's disease. She made an uneventful recovery with normalization of hematological and biochemical parameters without a specific treatment over a period of few weeks.

One year later, she was seen in the hospital with low-grade fever, malaise, loss of appetite, and loss of weight with cervical lymphadenopathy, and repeat biopsy of the cervical lymph nodes revealed similar histopathology compatible with Kikuchi's disease. She was managed conservatively, and the patient made a full recovery. Currently, she is asymptomatic and under follow-up.

## 3. Discussion

Kikuchi's disease is a benign condition which is more common among young females of tropics [1, 2]. It is mostly a self-limiting condition of lymphadenopathy mainly involving the cervical group of lymph nodes [5, 6]. It can be associated with other constitutional symptoms such as fever, loss of appetite, and loss of weight. Diagnosis of Kikuchi's disease is mainly based on the histological assessment of excised lymph node by an experienced histopathologist. Histological features in favor of Kikuchi's disease include fragmentation, necrosis, and karyorrhexis with paucity of granulocytes [15]. But it could be frequently misdiagnosed as a malignant lymphoma. Three histopathological types of Kikuch's disease are described which include proliferative, necrotizing, and xanthomatous type [16, 17].

Differential diagnosis of cervical lymphadenopathy is broad and exhausting which includes infections (infectious mononucleosis, cat scratch fever, toxoplasmosis, and tuberculosis), inflammatory conditions (SLE), or malignancies (lymphoma or metastatic) to name a few. So, the diagnosis of Kikuchi's disease should be made very cautiously after careful exclusion of these causes including the sinister ones. Kikuchi's disease can be a great masquerader of lymphoma [18].

Recurrence of lymphadenopathy in Kikuchi's disease is unusual. A study by Cheng et al. reported 14 patients (*n* = 195) having clinical recurrence after 6-month period [19]. But other studies report a lower incidence of recurrence of KFD after the 1^st^ episode which is estimated around 3% [20]. Blewitt reported a recurrence of Kikuchi's disease after 12 years following the initial episode [21]. Another interesting case study of a patient reports 4 relapses within a period of 18 years [22]. We report a confirmed case of Kikuchi's disease relapsing after one month of complete remission of symptoms and regression of lymph nodes. As the recurrence is rare in Kikuchi's disease, it is very prudent to exclude other sinister causes of lymphadenopathy in the later presentation. So, repeat biopsy and histological assessment is of utmost importance before labeling the patient with a recurrence of Kikuchi's disease. The etiopathogenesis of rare but reported recurrences in KFD disease is still to be elucidated, but we believe that underlying autoimmunity is responsible for the recurrent hits in KFD in susceptible individuals, and further studies are indeed warranted to hunt for a genetic or environmental trigger in this group.

Another interesting finding in this case report is the long-standing thrombocytopenia of around 100 × 10^6^/L platelets. She had been extensively investigated for her thrombocytopenia including ANA, RF, and anti-CCP, and no cause was found. But immune thrombocytopenia could be a part of an autoimmune spectrum disorders such as SLE which could evolve in the future. Santana et al. described that Kikuchi's disease patients are at increased risk of developing SLE at a later time [23].

During the two episodes of Kikuchi's disease, we observed that patient's platelet count increased to nadir of 150 × 10^6/^L. We believe the most likely explanation for this is thrombopoietin is an acute-phase reactant, and thrombocytosis is commonly observed in acute inflammatory conditions. It is further supported in our patient by the elevation of erythrocyte sedimentation rate and elevation of the ferritin level.

## 4. Conclusion

Recurrence of Kikuchi's disease is rare. It is prudent to confirm the recurrence histologically in diagnosis of relapsing Kikuchi's disease as it can masquerade other sinister diagnoses such as lymphoma.

## Figures and Tables

**Table 1:** The results of laboratory tests of the patient before the biopsy.

	Laboratory investigations	Results	Normal range (for middle-aged woman)
Hematology	Hemoglobin	11.1 g/dl	12–16 g/dl
	White blood cell	3100 per microliter	—
	Total leukocyte count	775/mm^3^	4000–10000/mm^3^
	Platelet count	145 × 10^9^/L	150–400 × 10^9^/L
	Red blood cell count	4.5 million/mm^3^	4.2–5.4 million/mm^3^
	Mean corpuscular volume	80 fl	77–97 fl
	Neutrophils	62%	42–75%
	Lymphocytes	25%	20–51%
	Erythrocyte sedimentation rate	76 mm/h	0–30 mm/h

Biochemical serology	Blood urea nitrogen	16 mg/dl	8–20 mg/dl
	Serum creatinine	1.2 mg/dl	0.6–1.1 mg/dl
	Total bilirubin	0.7 mg/dl	0.2–1.2 mg/dl
	Direct bilirubin	0.3 mg/dl	0.1–0.3 mg/dl
	Serum alanine transaminase	11 U/l	Up to 31 U/l
	Serum aspartate transaminase	22 U/l	Up to 31 U/l
	Serum alkaline phosphatase	286 U/l	64–306 U/l
	Random blood sugar	92 mg/dl	80–140 mg/dl
	Lactate dehydrogenase	525 U/L	100–500 U/L
	Ferritin	295 ng/mL	5–130 ng/mL
	Globulins	40 g/L	20–35 g/L
	Albumin	34 g/L	34–54 g/L

Serology	Rheumatoid factor	1 U/mL	Up to 14 U/mL
	Anti-CCP	6	Negative <20 U/mL
	Antinuclear antibody (ANA)	Negative	—
	Anti-ds DNA	Negative	—
	Hepatitis A, B, C ab	Negative	—
	Human immunodeficiency viruses	Negative	—
	Occult blood	Negative	—
	Anti-EBV ab Ig M	Negative	—
	Antitoxoplasmosis ab Ig M	Negative	—
	Anti-CMV AbIg M	Negative	—
	Hepatitis A, B, C ab	Negative	—

## Data Availability

Data sharing is not applicable to this article as no datasets were generated or analyzed during the current study.
